# Laxative Effects of Dietary Supplementation with Sujiaonori Algal Biomaterial in Japanese Adult Women with Functional Constipation: A Case Study

**DOI:** 10.3390/jfb8020015

**Published:** 2017-05-15

**Authors:** Nlandu Roger Ngatu, Mitsunori Ikeda, Hiroyuki Watanabe, Mamoru Tanaka, Masataka Inoue

**Affiliations:** 1 Graduate School of Health Sciences & Nursing, University of Kochi, Kochi 781-8515, Japan; mikeda@cc.u-kochi.ac.jp (Mi.I.); inoue@cc.u-kochi.ac.jp (Ma.I.); 2 Faculty of Nutrition, University of Kochi, Kochi 781-8515, Japan; watana@cc.u-kochi.ac.jp (H.W.); m-tanaka@cc.u-kochi.ac.jp (M.T.)

**Keywords:** constipation, dietary intervention, Sujiaonori, women

## Abstract

Constipation is a gastrointestinal motility disorder that represents a major health problem in Japan. Approximately 26% of young Japanese adult women are reported to have this complaint. We report on the health effects of daily intake of Sujiaonori algal biomaterial (SBM) on constipation on 12 Japanese adult women. Data are from a four-week dietary intervention study on the health effects of daily Sujiaonori supplementation on cardiovascular, skin, and gastrointestinal health in which 32 adult Japanese volunteers (age range: 20–54 years) participated. They underwent clinical and laboratory investigations, and completed two study questionnaires (the brief diet history questionnaire (BDHQ) and the current health questionnaire) before and after dietary intervention. Of the 12 women volunteers with functional constipation, there were six SBM-supplemented subjects who received 3 g of Sujiaonori powder twice daily during meal, whereas the six others (controls) were from the group of those who took 3 g of a power made of 70% corn starch and 30% Japanese spinach mixture. The analysis of data on daily nutrient intake showed no significant dietary changes for nutrients (minerals, proteins, fiber, fat) and calorie intake (except alcohol intake that was reduced) in both groups. In SBM group, a significant reduction of the proportion of women with constipation was observed (*p* < 0.001), whereas no significant change was noted within the control group (*p* > 0.05). When both groups were compared, SBM was more effective than the control product; 66.7% (4/6) of SBM-supplemented women had their constipation relieved, whereas only one control (16.7%) controls benefited from dietary intervention (*p* < 0.001). In addition, no adverse effect was reported in the SBM group, whereas two controls reported nausea at post-survey. These results suggest that Sujiaonori contains compounds that can improve gastrointestinal function and relieve constipation.

## 1. Introduction

Constipation is a gastrointestinal motility disorder characterized by infrequent bowel movements, difficulty during defecation, and sensation of incomplete bowel evacuation [1,2]. Functional constipation is a prevalent and burdensome disorder that can generate considerable abdominal pain and distension, anorexia, and nausea. Approximately 14% of adult population worldwide and, in the United States, it accounted for 3.2 million visits to medical centers in 2012 [3,4,5]. It is considered a major health problem in Japan and also in most western countries [6,7,8]. Regarding its etiology, functional constipation can be caused by a number of factors such as insufficient intake of dietary fiber, reduced physical activity, and insufficient fluid intake [6]. A study by Murakami and colleagues conducted among young Japanese women aged 18–20 years showed a prevalence of self-reported constipation of 26% [9].

In the literature, a number of plant biomaterials and food products have been reported to exhibit laxative properties by increasing the intestinal motility, frequency, and weight of stools, such as extract of *Aloe ferox Mill* [10], agarwood (*Aquillaria sinensis, Aquilaria crasna*) [11], *Liriope platyphylla* [2], and prunes [12]. Sujiaonori, the Japanese name for *Ulva (Enteromorpha) prolifera Müller* growing in Shimanto River and mass cultivated in farms within Kochi prefecture. This green river alga is edible and rich in fiber (60–65% dry weight) [13]; it possesses several bioactive properties such as antioxidant, anti-inflammatory, immunostimulant, and lipid lowering effects [14,15]. However, to our knowledge, there has been no reported study on the laxative effects of Sujiaonori or its compounds in the literature. We conducted a four-week dietary intervention on the health effects of daily Sujiaonori algal biomaterial (SBM) supplementation in a sample of Japanese women. Here, we report on the health effects of SBM and control product intake on functional constipation on functional constpation.

## 2. Materials and Methods

We report on the health effects of daily intake of SBM and the control product on constipation in 12 Japanese adult women. Data are from a four-week dietary intervention study involving 32 adult Japanese female volunteers aged 20–54 years who participated in a controlled investigator-blinded dietary intervention study on the health effects of daily Sujiaonori supplementation on cardiovascular, skin, and gastrointestinal health. Participants underwent clinical and laboratory investigations, and completed two study questionnaires (the brief diet history questionnaire, BDHQ, and current health questionnaire) before and after dietary intervention. Details on the methodology used, as well as the description of study questionnaires, have been reported previously [16,17].

Data contained in this report are from 32 female volunteers aged 20–54 years (including nursing students and staff from the University of Kochi, Kochi, Japan) who completed the study questionnaires, were analyzed. Two groups were created: SBM group (*N* = 16) whose subjects received twice 3 g of Sujiaonori powder twice daily during meal, and the control group (*N* = 16) whose subjects had to take the corresponding amount of a power made of 70% corn starch and 30% Japanese spinach mixture. A participant had to report functional constipation if she or he had fewer than three defecations in a week, associated with a sense of incomplete evacuation and hard stools in the previous month and when joining this study, without any major disease that would have caused obstruction of bowel transit. The report on the health effects of SBM supplementation on adiponectin production and cardiovascular health has been published elsewhere [16].

This study complied with the code of ethics of the World Medical Association (Declaration of Helsinki). The protocol of the main study was approved by the research ethics committee of the Faculty of Nutrition, University of Kochi, Japan, and registered at the International Standard Randomized of Clinical Trial Number registry (clinical trial registration No ISRCTN35616776) [17]. The brief self-administered diet history questionnaire (BDHQ), University of Tokyo [18] and the current health status questionnaire (CHQ), which was prepared by our research team, were anonymously answered before and after intervention by each of the study participants. Samples of dried Sujiaonori, spinach, and corn starch were purchased from local food manufacturers. Both supplements (SBM and control product) were processed at the food science laboratory, University of Kochi, and put in small packs without mentioning the name of the content. 

Data are presented as proportions, the presence (1) or the absence (0) of a gastrointestinal functional symptom at baseline and end-of-study considered as 100% and 0% in the analysis, respectively. Fisher’s exact test was used to compare the study groups. Stata software version 10 was used to perform the statistical analysis. *p*-values (double-sided) of less than 0.05 are considered significant.

## 3. Results

Table 1 shows baseline sociodemographic and clinical characteristics of the participants in the main study. There were 32 women who completed the study questionnaires and the four-week dietary supplementation, including 90.6% (29/32) university students and 9.4% (3/32) teaching staff (*p* > 0.05). The mean age was 22.5 ± 7.1 (23.2 ± 9 controls and 21.8 ± 4.5 for SBM group). Both study groups comprised mainly students (87–88%). All participants were nonsmokers; 75% (24/32) were taking alcohol, including 13 (81.3%) controls and 11 (68.8%) women from SBM group (*p* > 0.05). More than half of participants, 59.4% (19/32), were not exercising (Table 1).

Participants were also asked to report on the intake of algal food product; only one (6.3%) control has been taking it in the month preceding the study (Table 1). Regarding dietary intake of nutrients (deriving from the BDHQ questionnaire), total daily energy intake was 1315.6 ± 518.7 and 1480.8 ± 630.1 kcal in controls and SBM group, respectively; whereas total daily fiber intake was estimated to be 8.3 ± 4.1 and 8.5 ± 5.5 kcal, respectively. No significant difference was observed when comparing both groups regarding fiber and energy intake [16]. On the other hand, there were 37.5 (12/32) of participants who reported having a functional constipation, including 37.5% (6/16) of SBM-supplemented subjets and 37.5% (6/16) of controls at baseline. There were 6.3% (2/32) of constipated women from SBM group who reported dyspepsia or loss of appetite at baseline (Table 1). None (0%) of the constipated women was exercising neither before nor during the dietary intervention. In addition, none (0%) of them has been taking algal food product.

### Intake of Sujiaonori Algal Biomaterial Exerts Laxative Effect in Women with Baseline Constipation

**Figure 1:**
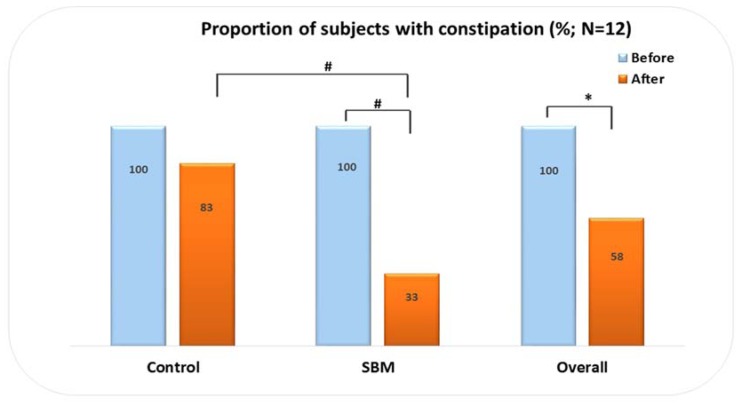
Proportion of women with constipation according to supplementation group. (***Legend***: #, *p*-value less than 0.001; *, *p*-value less than 0.05; SBM, Sujiaonori algal biomaterial. The figure shows a significant reduction of the proportion of women reporting constipation within SBM group (*p* < 0.001) and also when comparing SBM and control groups (*p* < 0.001)).

The overall results showed that 58.3% (7/12) of the 12 women who reported functional constipation at baseline still had this complaint at the end of study, whereas 41.6% (5/12) had their constipation relieved (*p* < 0.05). SBM was more effective than the control product; 66.7% (4/6) of SBM-supplemented women had their constipation relieved (*p* < 0.001), whereas a lower proportion of controls, 16.7% (1/6), benefited from the dietary intervention (*p* > 0.05). When both supplementation groups were compared, a significant difference was observed (*p* < 0.001) (Figure 1).

On the other hand, the two (100%) SBM-supplemented women who reported baseline dyspepsia and loss of appetite had these conditions relieved at the end of study (*p* < 0.001) (not shown). None of the SBM-supplemented women reported any side-effects; however, two of the six control women reported either loss of appetite or diarrhea. This suggests that they might have reacted to one of the components in the control supplement, corn starch or Japanese spinach.

## 4. Discussion and Conclusions

Constipation is a common complaint and its prevalence increases with age [19]. Our study showed that the dietary intake of SBM supplement relieved this gastrointestinal symptom in a sample of Japanese women. The overall prevalence of constipation among the main study participants was 37.5%, which is higher compared to rates reported previously. Another study showed a prevalence of self-reported constipation of 26% among young Japanese young adult women [9]. In North America, rates of 26% and 28% have been reported in women and the general population, respectively [19,20].

The present study showed that, of the six SBM-supplemented women who had functional constipation at baseline, four (approximately 67%) had this symptom relieved (vs. one out of six (17%) in controls). Sujiaonori is a river algal species growing in Kochi prefecture, Japan. *Ulva prolifera* is a nutritious food containing proteins, unsaturated fatty acids, polyphenols and minerals. Moreover, it is rich in fiber, representing approximately 60% of compounds of this this river alga [21]. By taking 3 g of SBM twice a day, subjects were being daily supplemented with an estimated 3.6 g of fiber, which might have balanced the diet and induced the improvement of gastrointestinal transit and the relief of constipation.

Dietary fiber is reported to regulate gastrointestinal transit time [22] and, according to a work by Lembo and Camillieri, increasing fiber (fruits, vegetables, or fiber supplement) intake is recommended as a primary measure in the treatment of constipation [23]. Algal fiber has been used for the treatment of various gastrointestinal disorders (including constipation, diarrhea, and ulcerative colitis) [24,25,26] and other conditions such as hypercholesterolemia and obesity [27,28]. On the other hand, physical exercise is known to influence gastrointestinal function and can relieve constipation. In our study, no difference was found in terms of exercise when comparing the pre- and post-survey status in both Sujiaonori and control groups. In addition, no association was found between physical exercise and dietary intake of supplement. Therefore, considering the results from this preliminary report, Sujiaonori could possibly be a candidate natural remedy for functional gastrointestinal disorders, particularly for constipation.

Nonetheless, this report is limited by fact that it provides information on the beneficial effects of SBM on functional constipation but in a few numbers of subjects. In addition, constipation was not the primary outcome variable of the study. Thus, conducting a clinical trial in which only subjects with functional constipation will take part should be envisaged.

In conclusion, this dietary intervention trial showed that SBM relieved functional constipation in women who had this gastrointestinal motility disorder prior to getting supplemented, suggesting that Sujiaonori—a fiber-rich green river alga that grows in Japan—contains compounds that improve gastrointestinal motility and relieve constipation. Further research is needed to confirm findings from this study.

## Figures and Tables

**Table 1:** Sociodemographic and Clinical Characteristics of Participants at Baseline.

Social, Clinical, and Lifestyle-Related Characteristics of Participants	Overall (*N* = 32)	Control (*N* = 16)	SBM (*N* = 16)
Occupation [*N* (%)]			
Student	29 (90.6)	14 (87.5)	14 (87.5)
Staff	3 (9.4)	2 (12.5)	2 (12.5)
Total	32 (100)	16 (100)	16 (100)
Age (years; mean ± SD)	22.5 ± 7.1	23.2 ± 9	21.8 ± 4.5
Tobacco Smoking [*N* (%)]			
Yes	0 (0)	0 (0)	0 (0)
No	32 (100)	16 (51.5)	16 (48.5)
Alcohol Consumption [*N* (%)]			
Yes	24 (75)	13 (81.3)	11 (68.8)
No	8 (25)	3 (18.7)	5 (31.2)
Physical Exercise [*N* (%)]			
Not at all	19 (59.4)	10 (62.5)	9 (56.2)
Once a week at least 20 min	1 (3.1)	0 (0)	2 (12.5)
Twice a week at least 20 min	4 (12.5)	2 (12.5)	2 (12.5)
Three times or more a week, 20 min	8 (25)	4 (25)	3 (18.8)
Intake of Algal Product [*N* (%)]			
Yes	1 (3.1)	1 (6.3)	0 (0)
No	31 (96.9)	15 (93.8)	16 (100)
Functional Gastrointestinal Disorder [*N* (%)]			
Constipation	12 (37.5)	6 (37.5)	6 (37.5)
Others (dyspepsia, appetite loss, diarrhea)	2 (6.3)	0 (0)	2 (12.5)

*N*, number of participants; %, percentage; SD, standard deviation.

## References

[1] Mc Cullum I.J.D., Ong S., Mercer-Jones M. (2009). Chronic constipation in adults. BMJ.

[2] Kim J.E., Lee Y.J., Kwak M.H., Ko J., Hong J.T., Hwang D.Y. (2013). Aqueous extracts of Liriope platyphylla induced significant laxative effects on loperamide-induced constipation of SD rats. BMC Complement. Altern. Med..

[3] Cirillo C., Capasso R. (2015). Constipation and Botanical Medicines: An Overview. Phytother. Res..

[4] Peery A.F., Dellon E.S., Lund J., Crockett S.D., McGowan C.E., Bulsiewicz W.J., Gangarosa L.M., Thiny M.T., Stizenberg K., Morgan D.R. (2012). Burden of gastrointestinal disease in the United States: 2012 update. Gastroenterology.

[5] Dimidi E., Christodoulides S., Fragkos K.C., Scott S.M., Whelan K. (2014). The effect of probiotics on functional constipation in adults: a systematic review and meta-analysis of randomized controleed trials. Am. J. Clin. Nutr..

[6] Thompson W.G., Longstreth G.F., Drossman D.A., Heaton K.W., Irvine E.J., Muller-Lissner S.A. (1999). Functional bowel disorders and functional abdominal pain. Gut.

[7] Hirai K., Higuchi H., Sato R., Kitano N., Furusaki K., Takezoe R., Okada S., Ogoshi K. (2001). Awareness of the health and defecation tendencies among college students by location of domicile. Jpn. J. Hyg..

[8] Nellesen D., Yee K., Chawla A., Lewis B.E., Carson R.T. (2013). A systematic review of the economic and humanistic burden of illness in irritable bowel syndrome and chronic constipation. J. Manag. Care Pharm..

[9] Murakami K., Ohkubo H., Sasaki S. (2006). Dietary intake in relation to self-reported constipation among Japanese women aged 18–20 years. Eur. J. Clin. Nutr..

[10] Wintola O.A., Sunmonu T.O., Afolayan A.J. (2010). The effect of Aloe ferox Mill in the treatment of loperamide-induced constipation in Wistar rats. BMC Gastroenterol..

[11] Hara H., Ise Y., Morimoto N., Shimazawa M., Ichihashi K., Ohyama M., Iinuma M. (2008). Laxative effect of agarwood and its mechanism. Biosci. Biotechnol. Biochem..

[12] Stacewicz-Sapuntzakis M., Bowen P.E., Hussain E.A., Damayanti-Wood B.I., Farnsworth N.R. (2001). Chemical composition and potential health effects of prunes: A functional food?. Crit. Rev. Food Sci. Nutr..

[13] Ngatu N.R., Ikeda M., Kanbara S., Inoue M., Suzuki M., Watanabe H., Umebara M. (2015). Potential health effects of green river algae (Aonori) of the LPP complex, with a reference to *Ulva prolifera*. Int. J. Gen. Med..

[14] Patel S. (2012). Therapeutic importance of sulfated polysaccharides from seaweeds: Updating the recent findings. 3 Biotech.

[15] Tang Z., Gao H., Wang S., Wen S., Oin S. (2013). Hypolipidemic and antioxidant properties of a polysaccharide fraction from Enteromorpha prolifera. Int. J. Biol. Macromol..

[16] Ngatu N.R., Ikeda M., Watanabe H., Tanaka M., Inoue M., Kanbara S., Nojima S. (2017). Uncovering adiponectin replenishing property of Sujiaonori algal biomaterial in humans. Mar. Drugs.

[17] International Standard Randomised Clinical Trial Number (ISRCTN) (2015). Health effects of dietary supplementation of sujiaonori biomaterial on adiponectin, cardiovascular health parameters and skin health in humans. BMC.

[18] Sakata S., Tsuchihashi T., Oniki H., Tominaga M., Arakawa K., Sasaki M., Kitazono T. (2015). Relationship between salt intake as estimated by a brief self-administered diet-history questionnaire (BDHQ) and 24-h urinary salt excretion in hypertensive patients. Hypertens. Res..

[19] Schuster B.G., Kosar L., Kamrul R. (2015). Constipation in older adults. Can. Fam. Physician.

[20] Higgins P.D., Johanson J.F. (2004). Epidemiology of constipation in North America: A systematic review. Am. J. Gatsroenterol..

[21] Aguilera-Morales M., Casas-Valdez M., Carrillo-Dominguez S., Gonzalez-Acosta B., Perez-Gil F. (2005). Chemical composition and microbiological assays of marine algae Enteromorpha spp. as a potential food source. J. Food Compos. Anal..

[22] Raposo M.F.J., Morais A.M.M.B., Morais R.M.S.C. (2016). Emergent sources of prebiotics: Seaweeds and microalgae. Mar. Drugs.

[23] Lembo A., Camilleri M. (2003). Chronic constipation. N. Engl. J. Med..

[24] McRorie J.W., Daggy B.P., Morel J.G., Diersing P.S., Miner P.B., Robinson M. (1998). Psylium is superior to docusate sodium for treatment of chronic constipation. Aliment. Pharmacol. Ther..

[25] Mehmood M.H., Aziz N., Ghayur M.N., Gilani A.H. (2011). Pharmacological basis for the medicinal use of psyllium husk (Ispaghula) in constipation and diarrhea. Dig. Dis. Sci..

[26] Fernandez-Banares F., Hinojosa J., Sanchez-Lombrana J.L., Navarro E., Martinez-Salmeron J.F., Garcia-Puges A., Gonzalez-Huix F., Riera J., Gonzalez-Lara V., Dominguez-Abascal F. (1999). Randomized clinical trial of Plantago ovata seeds (dietary fiber) as compared with mesalamine in maintaining remission in ulcerative colitis. Spanish group for the study of Crohn's disease and ulcerative colitis (GETECCU). Am. J. Gastroenterol..

[27] Moreyra A.E., Wilson A.C., Koraym A. (2005). Effect of combining psyllium fiber with simvastatin in lowering cholesterol. Arch. Intern. Med..

[28] Salas-Salvado J., Farres X., Luque X., Narejos S., Borreli M., Basora J., Anguera A., Torres F., Bullo M., Balanza R. (2008). Effect of two doses of a mixture of soluble fibres on body weight and metabolic variables in overweight or obese patients: A randomised trial. Br. J. Nutr..

